# Engineering the Interfacial Microenvironment via Surface Hydroxylation to Realize the Global Optimization of Electrochemical CO_2_ Reduction

**DOI:** 10.1021/acsami.2c09129

**Published:** 2022-07-11

**Authors:** Xu Han, Ting Zhang, Martí Biset-Peiró, Xuan Zhang, Jian Li, Weiqiang Tang, Pengyi Tang, Joan Ramon Morante, Jordi Arbiol

**Affiliations:** † Catalan Institute of Nanoscience and Nanotechnology (ICN2), CSIC and BIST, Campus UAB, Bellaterra, Barcelona, 08193 Catalonia, Spain; ‡ Catalonia Institute for Energy Research (IREC), Jardins de les Dones de Negre 1, Sant Adrià del Besòs,Barcelona, 08930 Catalonia, Spain; § Department of Materials Engineering, KU Leuven, 3001 Leuven, Belgium; ∥ Laboratory of Renewable Energy Science and Engineering, Institute of Mechanical Engineering EPFL, Station 9, 1015 Lausanne, Switzerland; ⊥ State Key Laboratory of Chemical Engineering and School of Chemical Engineering, East China University of Science and Technology, 200237 Shanghai, China; # State Key Laboratory of Information Functional Materials, 2020 X-Lab, Shanghai Institute of Microsystem and Information Technology, Chinese Academy of Sciences, 200050 Shanghai, China; ¶ Department of Physics, Universitat de Barcelona, Barcelona, 08028 Catalonia, Spain; ∇ ICREA, Pg. Lluís Companys 23, Barcelona, 08010 Catalonia, Spain

**Keywords:** ZnO, surficial hydroxyls, CO_2_ adsorption, CO_2_ activation, metal−organic frameworks (MOFs)

## Abstract

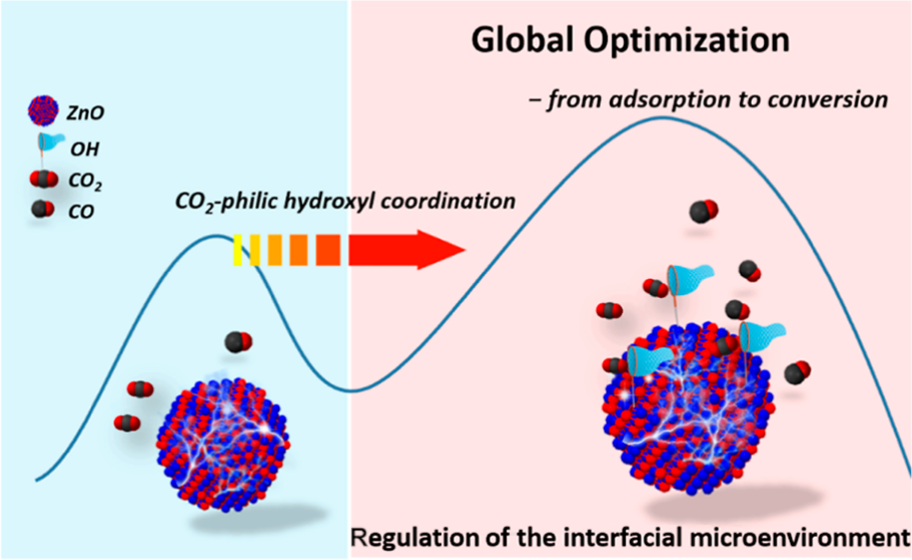

The adsorption and activation of CO_2_ on the electrode interface is a prerequisite and key step for electrocatalytic CO_2_ reduction reaction (eCO_2_ RR). Regulating the interfacial microenvironment to promote the adsorption and activation of CO_2_ is thus of great significance to optimize overall conversion efficiency. Herein, a CO_2_-philic hydroxyl coordinated ZnO (ZnO–OH) catalyst is fabricated, for the first time, via a facile MOF-assisted method. In comparison to the commercial ZnO, the as-prepared ZnO–OH exhibits much higher selectivity toward CO at lower applied potential, reaching a Faradaic efficiency of 85% at −0.95 V versus RHE. To the best of our knowledge, such selectivity is one of the best records in ZnO-based catalysts reported till date. Density functional theory calculations reveal that the coordinated surficial −OH groups are not only favorable to interact with CO_2_ molecules but also function in synergy to decrease the energy barrier of the rate-determining step and maintain a higher charge density of potential active sites as well as inhibit undesired hydrogen evolution reaction. Our results indicate that engineering the interfacial microenvironment through the introduction of CO_2_-philic groups is a promising way to achieve the global optimization of eCO_2_ RR via promoting adsorption and activation of CO_2_.

## 1 Introduction

The electrochemical CO_2_ reduction reaction (eCO_2_ RR) into various fuels and value-added chemicals is a promising method to eliminate excessive greenhouse gas and realize energy reuse toward carbon recycling.^1−4^ Considering the products of eCO_2_ RR, carbon monoxide (CO), an important raw material for top-level organic chemical products, has high industrial value.^5−7^ Theoretically, CO_2_-to-CO conversion goes through the following steps: (1) adsorption of CO_2_ and activation through a proton-coupled electron transfer process to generate COOH* intermediates; (2) the adsorbed COOH* intermediate is further reduced to form CO* and water; and (3) CO* is desorbed from the surface of the catalyst to form the CO product.^8−12^ Due to the poor solubility of CO_2_ in the aqueous electrolyte, the transformation of CO_2_ from the gas feed to the surface of active sites is a minimum prerequisite for the follow-up steps of eCO_2_ RR, thus limiting the overall conversion efficiency.^13,14^

Zinc oxide (ZnO), characterized by its huge reserves and for being cost-friendly, has been widely investigated for generating CO with moderate selectivity.^15−21^ The oxidation state of Zn in ZnO as a clear active site provides infinite possibilities to enhance the eCO_2_ RR efficiency.^18−21^ For example, an increased number of active sites could be induced by modulating the ZnO morphology to expose abundant edge facets;^17^ the ratio of H_2_/CO obtained on ZnO electrocatalysts could be tuned through controlling the defects and facets.^19^ However, almost no attention has been paid to the interfacial microenvironment between ZnO catalysts and CO_2_, a key factor to affect its adsorption and activation. Generally, the adsorption and activation of non-polar CO_2_ occur only at the interface of the solid electrocatalyst with the liquid electrolyte and CO_2_ molecules by weak interactions.^11,22−25^

Introducing carbon dioxide-philic functional groups, which have strong interaction with CO_2_ molecules, is an appealing route to manipulate the interface to enhance CO_2_ affinities.^11,26−30^ It is also anticipated that the CO_2_-philic functional groups on the surface could modulate the electronic structure of the catalyst to further manipulate the formation of the sequent intermediates in the CO_2_-to-CO conversion.^11,29^ With this in mind, −OH groups, a kind of CO_2_-philic functional groups, for the first time, were introduced on the surface of ZnO catalysts (ZnO–OH) via a simple ZIF-8-assisted (ZIF stands for zeolitic imidazolate frameworks) method. Compared to the commercial ZnO, the ZnO–OH exhibited much higher selectivity toward CO at a relatively lower applied potential and reached a FE_CO_ maximum of 85% at −0.95 V versus RHE, which is one of the best values among ZnO-based catalysts reported till date (see Table S4). Density functional theory (DFT) calculations indicated the existence of strong attraction between the ZnO–OH and the CO_2_ molecule, which is beneficial to the adsorption of CO_2_. Furthermore, the hydroxyl groups play an important role in facilitating the formation of the follow-up intermediates (COOH* and CO*), simultaneously limiting the undesired hydrogen evolution reaction (HER). All the results reveal the crucial role of CO_2_-philic −OH groups in promoting the interfacial adsorption and activation of CO_2_ to realize the global optimization of CO_2_ electroreduction, which benefits the understanding of the relevant mechanism in eCO_2_ RR and rational design of future high-efficient electrocatalysts.

## 2 Experimental Section

### 2.1 Chemicals

If not specified, all chemical reagents were obtained from Sigma-Aldrich and used directly. Zinc nitrate hexahydrate [Zn(NO_3_)_2_·6H_2_O], 2-methylimidazole (2-mim), commercial zinc oxide (ZnO), ethanol, and sodium bicarbonate (NaHCO_3_) were all of analytical grade. The Nafion membrane (N-117, 0.18 mm thick) and carbon paper were obtained from Alfa Aesar.

### 2.2 Material Preparation

#### 2.2.1 Preparation of ZIF-8

The fabrication process of ZIF-8 is similar to the process reported in the literature.^31^ 50 mL of methanol aqueous solution containing 2-mim (1.230 g) was added into the methanol aqueous solution of Zn(NO_3_)_2_·6H_2_O (50 mL, 1.115 g) with magnetic stirring to form homogeneous solution. After 24 h reaction without stirring at room temperature, the white solid was then taken out, washed by methanol several times, and vacuum dried overnight.

#### 2.2.2 Preparation of Zn_5_(OH)_8_(NO_3_)_2_(H_2_O)_2_

100 mg of ZIF-8 was etched by immersing into an ethanol solution (100 mL) containing 0.5 g of Zn(NO_3_)_2_·6H_2_O with stirring for 30 min. The obtained Zn_5_(OH)_8_(NO_3_)_2_(H_2_O)_2_ sample was then taken out, washed with ethanol, and dried in vacuum oven overnight.

#### 2.2.3 Preparation of ZnO–OH

The as-prepared Zn_5_(OH)_8_(NO_3_)_2_(H_2_O)_2_ powders were put at the porcelain boat. Subsequently, the samples were placed in a tube furnace and heated at 400 °C in air for 90 min to yield ZnO–OH (10 °C/min). Similarly, L-ZnO-OH and H–ZnO–OH were prepared by pyrolysis of Zn_5_(OH)_8_(NO_3_)_2_(H_2_O)_2_ that were treated by 0.4 or 0.6 g Zn(NO_3_)_2_·6H_2_O, respectively.

#### 2.2.4 Preparation of D–ZnO

The as-prepared ZIF-8 powders were put at the porcelain boat with a direct pyrolysis at 400 °C for 90 min to yield D–ZnO.

### 2.3 Ink Preparation

5 mg of the powder sample and 100 μL of Nafion solutions (5 wt %) were dissolved in 1 mL of ethanol under ultrasonication to form a homogeneous solution. 500 μL of the above inks were dropped onto the surface of the carbon paper (1 × 1 cm^2^). The loading mass of the catalyst was determined as ∼3 mg/cm^2^.

### 2.4 Electrochemical Characterization

The electrocatalytic performance was characterized in a three-electrode H-type cell system with two-compartments separated by a Nafion N-117 membrane, including a reference electrode (Ag/AgCl electrode), a counter electrode (Pt plate), and a working electrode (catalyst-loaded carbon paper). All potentials were referred to the RHE with *E*_RHE_ = *E*^0^_Ag/AgCl_ + *E*_Ag/AgCl_ + 0.059 × pH.^32,33^ A BioLogic VMP3 electrochemical workstation was used to perform electrochemical experiments.

During electrochemical CO_2_ reduction experiments, the CO_2_ gas was delivered with a rate of 20 mL min^–1^ into the cell, and gas chromatography was used to test the final gas phase composition every 20 min. Meanwhile, we collected data three times to get an average value.

## 3 Results and Discussion

### 3.1 Characterization of the Sample

In order to understand the influence of the −OH group on the CO_2_ adsorption, DFT was first used to calculate the free energy of CO_2_ adsorption on two representative models for ZnO and ZnO–OH (Figure S1). Compared to the negligible adsorption Gibbs free energy of CO_2_ molecule on the pristine ZnO (−0.0028 eV), a much larger adsorption energy of −0.1466 eV was observed on ZnO–OH, revealing that the CO_2_ adsorption on the ZnO–OH is more feasible. The increase in the CO_2_ adsorption affinity is beneficial for the following eCO_2_ RR and, in parallel, inhibits the reduction of protons (hydrogen evolution) in the electrolyte.^26,29^

**Figure 1:**
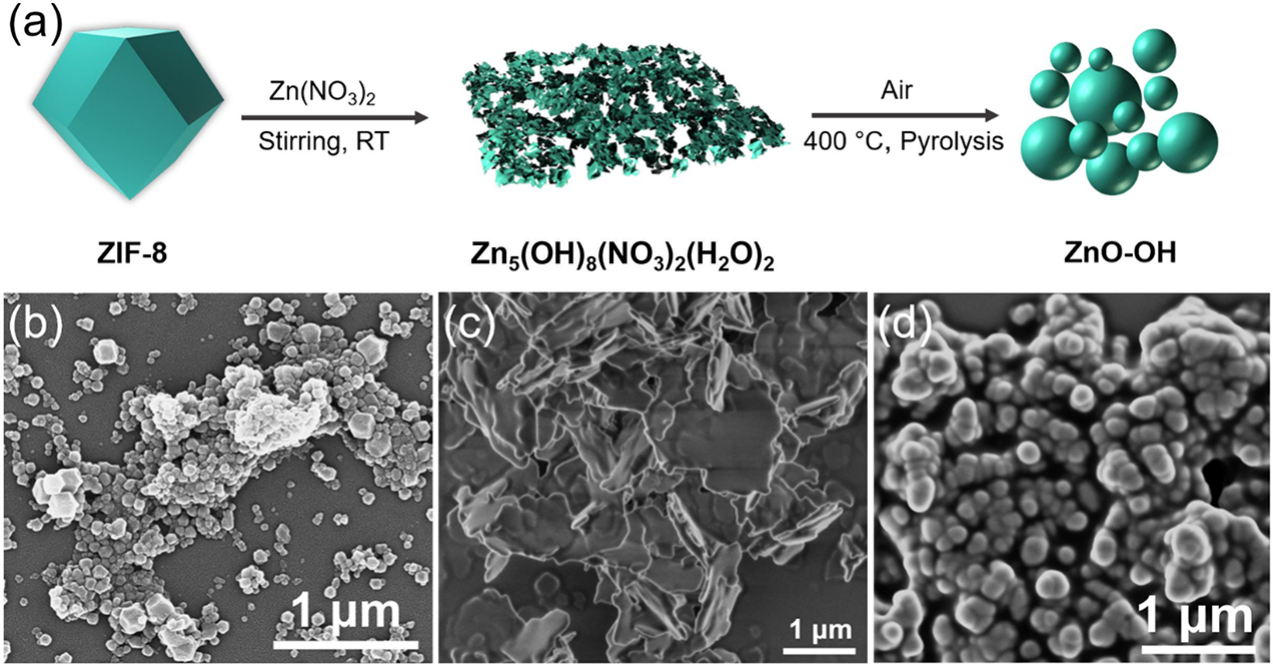
(a) Schematic illustration for the formation process of the ZnO–OH. SEM image of (b) ZIF-8, (c) Zn_5_(OH)_8_(NO_3_)_2_(H_2_O)_2_ and (d) ZnO–OH.

With the guidance of above DFT calculations, ZnO with rich surficial −OH was synthesized via a novel MOF-assisted procedure (Figure 1a). In brief, ZIF-8 as a precursor was first transformed into a hydroxide intermediate by virtue of adding a given amount of Zn(NO_3_)_2_ solution at room temperature. Afterward, the ZnO with rich surficial −OH (ZnO–OH) was obtained through pyrolysis of the above hydroxide intermediate under air. The high crystallinity of the as-prepared ZIF-8 and the corresponding hydroxide intermediate were first confirmed by powder X-ray diffraction (XRD) measurements. The ZIF-8 samples exhibited similar crystal patterns as expected for the standard ZIF-8 structure (Figure S2a).^31,34^ In the case of the hydroxide intermediate sample, new diffraction peaks belonging to the Zn_5_(OH)_8_(NO_3_)_2_(H_2_O)_2_ phase appeared, indicating that the treatment changed the crystal structure of the pristine ZIF-8 (Figure S2b).^35,36^ Field emission scanning electron microscopy revealed that sheet-shaped nanostructures were formed for the Zn_5_(OH)_8_(NO_3_)_2_(H_2_O)_2_ sample, different from the characteristic rhombic dodecahedral morphology of the ZIF-8 sample (Figures 1b,c and S3). Elemental composition maps indicated the existence of an additional O signal in the Zn_5_(OH)_8_(NO_3_)_2_(H_2_O)_2_ sample (Figures S4 and S5), while high-resolution TEM (HRTEM) analyses (Figure S6) further proved that ZIF-8 successfully transformed into the Zn_5_(OH)_8_(NO_3_)_2_(H_2_O)_2_ structure.

**Figure 2:**
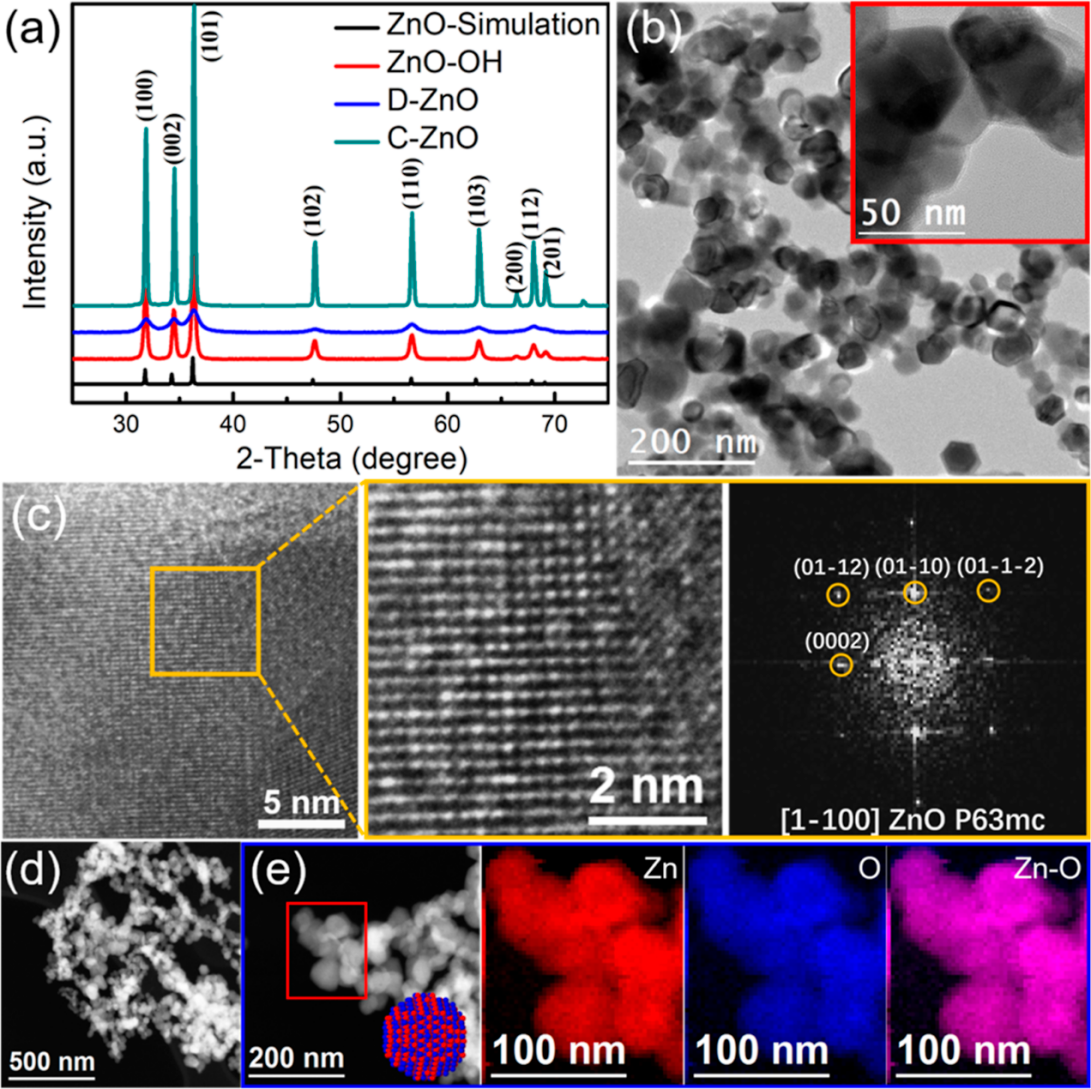
(a) XRD patterns. (b) Bright field TEM images showing the morphology of the ZnO–OH sample. The inset corresponds to a magnified detail of the studied area; (c) HRTEM image (left) and corresponding magnified detail (middle) with the corresponding indexed fast Fourier transform spectrum (right); (d) high-angle annular dark field scanning transmission electron microscopy (HAADF STEM) image; (e) HAADF STEM image and representative EELS chemical composition maps obtained from the red squared area in the STEM micrograph. Individual maps obtained from the Zn L_2,3_-edges at 1020 eV (red), O K-edges at 532 eV (blue), and Zn–O composite map. Inset in (e) shows a 3D atomic supercell model of a ZnO nanoparticle (Zn and O atoms are represented in red and blue, respectively).

In order to clarify the uniqueness of the ZnO–OH obtained by our method, a reference D–ZnO sample was synthesized through direct pyrolysis under air of an as-prepared ZIF-8 sample without Zn(NO_3_)_2_ treatment. Meanwhile, commercial ZnO was also used as a reference sample and was labeled as C–ZnO. As shown in Figure 2a, all samples clearly showed a similar diffraction pattern with that of simulated ZnO, indicating the successful synthesis of the ZnO skeleton.^18,19^ To investigate the morphology and phase evolution processes, SEM and TEM images of the three ZnO-based samples were obtained (Figures 1d, 2 and S7–10). The as-prepared ZnO–OH and the corresponding ZnO-based samples exhibited similar quasi-spherical shapes with irregular sizes. HRTEM analyses showed that all the ZnO-based samples displayed the typical hexagonal wurtzite ZnO phase (space group = *P*6_3_/*mmc*) with *a* = *b* = 3.2900 Å and *c* = 5.3000 Å.^37−40^ Electron energy loss spectroscopy (EELS) demonstrated the uniform distributions of Zn and O throughout all samples.

**Figure 3:**
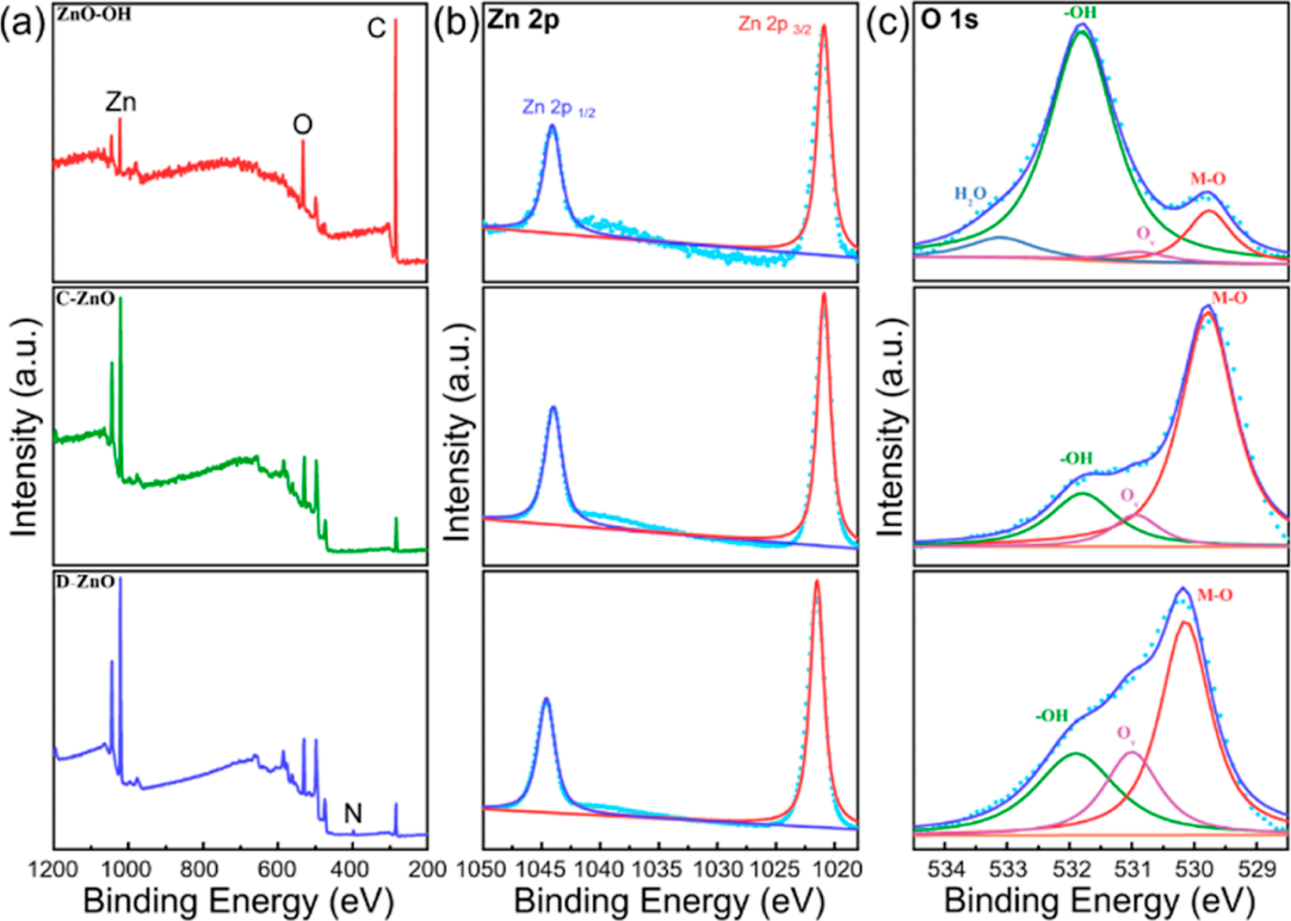
(a) XPS survey spectra and (b) high-resolution XPS spectra of Zn 2p and (c) O 1s for ZnO–OH (top), C–ZnO (middle), and D–ZnO (bottom).

X-ray photoelectron spectroscopy (XPS) was used to characterize the chemical valence state and surface compositions of the different catalysts. Figure 3a shows the presence of C, O, and Zn elements in all samples. The Zn 2p XPS core level spectra for all samples can be deconvoluted into two peaks, corresponding to Zn 2p_3/2_ and Zn 2p_1/2_ centered at around 1021 and 1044 eV, respectively, which indicates the presence of Zn^2+^ in all samples (Figure 3b).^19,31^ The high-resolution XPS spectra obtained on the O 1s show three peaks in all three samples, corresponding to the typical metal–oxygen bond (Zn–O, 529.8 eV),^18,19^ oxygen vacancy (O_v_, 531.0 eV),^41^ and −OH (531.7 eV),^42−44^ respectively (Figure 3c). It is worth noting that there is a significant signal enhancement of the corresponding −OH peak obtained on the ZnO–OH sample in comparison to the C–ZnO and D–ZnO, which is ascribed to the higher density of surficial −OH groups. The integral-area ratios of the peak of surficial −OH groups to the peak of oxygen vacancy were calculated to be 51.3, 2.1, and 1.3 for ZnO–OH, C–ZnO, and D–ZnO. In comparison to C–ZnO, the much stronger FTIR peaks at around 1500 and 3400 cm^–1^ for ZnO–OH (Figure S11), which are attributed to the O–H stretch mode,^45^ further confirmed the existence of large amount of −OH groups on the surface of ZnO–OH. All these observations demonstrated that the prepared ZnO–OH maintained the basic skeleton of ZnO with large amount of −OH groups on the surface. CO_2_ adsorption measurements on different samples were further performed to support the strong interaction between −OH groups and CO_2_ experimentally (Figure S12). The higher CO_2_ uptake is observed on ZnO–OH sample, with the order following ZnO–OH (15.44 m^2^/g) > D–ZnO (5.93 m^2^/g) > C–ZnO (1.64 m^2^/g). Such results demonstrated that the surface −OH groups of ZnO–OH promoted CO_2_ adsorption remarkably, consistent with the DFT results (Figure S1).

### 3.2 eCO_2_ RR Performance

**Figure 4:**
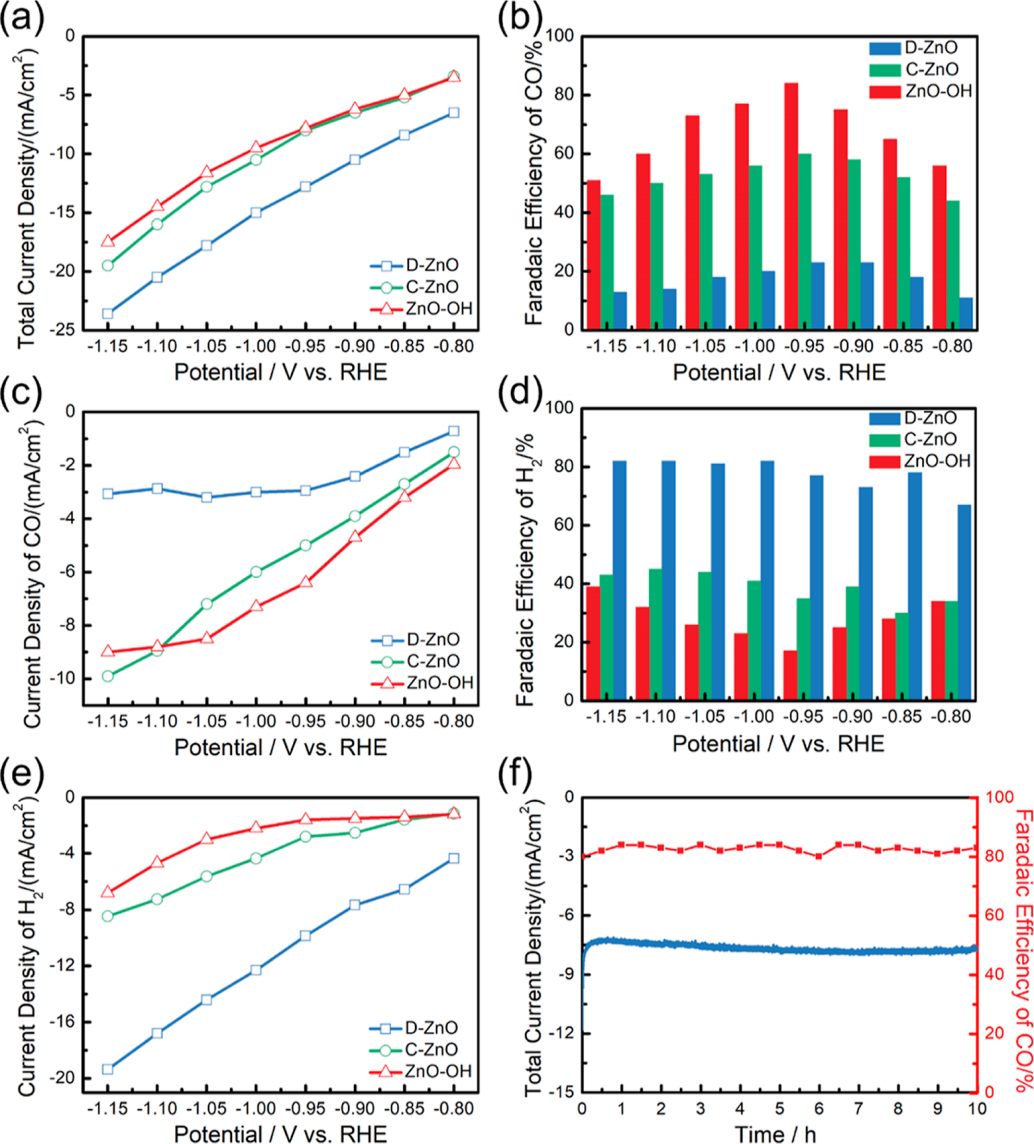
Electrocatalytic performance tests for ZnO–OH, D–ZnO and C–ZnO. (a) Total current density at various potentials. (b) FE of CO. (c) Current density of CO and (d) FE of H_2_. (e) Current density of H_2_ on ZnO–OH, D–ZnO, and C–ZnO. (f) Stability test for the ZnO–OH catalyst at −0.95 V vs RHE.

Next, electrochemical CO_2_ RR performances of the different samples were evaluated in 0.5 M NaHCO_3_. All electrodes were pre-treated in an Ar-saturated electrolyte at −0.70 V versus RHE for 30 min to reach a stable current (Figure S13). The eCO_2_ RR performance on the ZnO–OH sample was first evaluated by linear sweep voltammetry in both Ar- and CO_2_-saturated electrolytes (Figure S14). A large increase in the current density observed on the ZnO–OH sample after replacement of an Ar atmosphere by CO_2_ suggested that CO_2_ was electrochemically reduced by the ZnO–OH sample.^46,47^ Meanwhile, no obvious redox peaks were observed in the CO_2_-saturated aqueous solution, which displayed that ZnO–OH tended to react with CO_2_ molecules instead of suffering from self-reduction.^26^Figure 4a summarizes the measured total current density for ZnO–OH, D–ZnO, and C–ZnO samples. Although ZnO–OH and C–ZnO showed similar total reduction current densities, ZnO–OH showed the highest selectivity toward CO in a potential range from −0.80 to −1.15 V versus RHE, reaching the maximum Faradaic efficiency (FE) (CO) (85%) at −0.95 V versus RHE (Figure 4b). To the best of our knowledge, such high selectivity for CO at a low applied potential is the best record in ZnO-based catalysts reported so far (Table S4). The largest potential-dependent CO partial current densities observed on ZnO–OH further demonstrated the excellent activity and selectivity toward CO (Figure 4c). A decreasing trend in FE (CO) for ZnO–OH and C–ZnO was observed when the potential shifted to more negative values, which mainly stems from the dominance of the H_2_ generation over the eCO_2_ RR (Figure 4d). This assumption was further confirmed by the potential-dependent H_2_ current densities for the different catalysts (Figure 4e). The intrinsic activity of the catalysts was disclosed by the electrochemical active surface area (ECSA).^32^ As shown in Figure S15, the *C*_dl_ of ZnO–OH, D–ZnO, and C–ZnO samples was 0.05, 0.07, and 0.02 μF cm^–2^, respectively, which indicated that the ECSA is not responsible for the activity of ZnO–OH. A similar phenomenon could be observed on the nitrogen adsorption–desorption isotherms and BET surface area (Figure S16). It is well known that those electrocatalysts with high specific surface area should endow the efficient exposure of electrocatalytic active sites, fast electrolyte penetration/diffusion, and free diffusion of intermediates.^48,49^ In our case, the specific surface area of the ZnO–OH was much lower than that of D–ZnO, which suggests that the intrinsic catalytic activity of ZnO–OH sample arise from the presence of surficial −OH groups instead of the specific surface area.

To investigate the reaction kinetics on ZnO–OH during eCO_2_ RR, Tafel slopes derived from the static state current densities for CO were calculated. The C–ZnO samples exhibit a Tafel slope of 36 mV dec^–1^, close to 39 mV dec^–1^, indicating that the rate-determining step (RDS) of CO_2_ RR on C–ZnO powder corresponds to the initial proton-coupled electron transfer (Figure S17).^50^ The much lower Tafel slopes (30 mV dec^–1^) for the ZnO–OH catalyst indicated its remarkably improved kinetics toward CO conversion.^51−55^ In addition, the effects of ZnO–OH samples treated by different amounts of Zn(NO_3_)_2_ on the CO_2_ RR activity were also studied, which were denoted as L–ZnO–OH and H–ZnO–OH, respectively. Both the referential samples showed similar crystal patterns with simulated ZnO (Figure S18a) and morphology in comparison with spherical ZnO–OH (Figure S18b,c). However, the selectivity of L–ZnO–OH and H–ZnO–OH changed negatively (Figure S19), which is due to the decreased ratio between surficial −OH groups and oxygen vacancy (Figure S20).

To further investigate the stability of the ZnO–OH during the eCO_2_ RR, a 10 h stability measurement was conducted. A current density of ca. −8.2 mA cm^–2^ and a FE(CO) over 80% were maintained during the 10 h test (Figure 4f). After the stability test, TEM analyses were performed to reveal the morphology and phase changes on the ZnO–OH sample, as shown in Figure S21. EELS compositional maps demonstrate that most of the ZnO–OH areas showed a uniform distribution of Zn and O. HRTEM analyses showed the presence of some metallic Zn nanoparticles with hexagonal phase (space group = *P*6_3_/*mmc*). The presence of reduced Zn nanostructure can explain the slight efficiency loss after the stability test, evidencing the competition in the metal oxides between self-reduction and CO_2_ reduction.^26,56^

### 3.3 DFT Calculations

**Figure 5:**
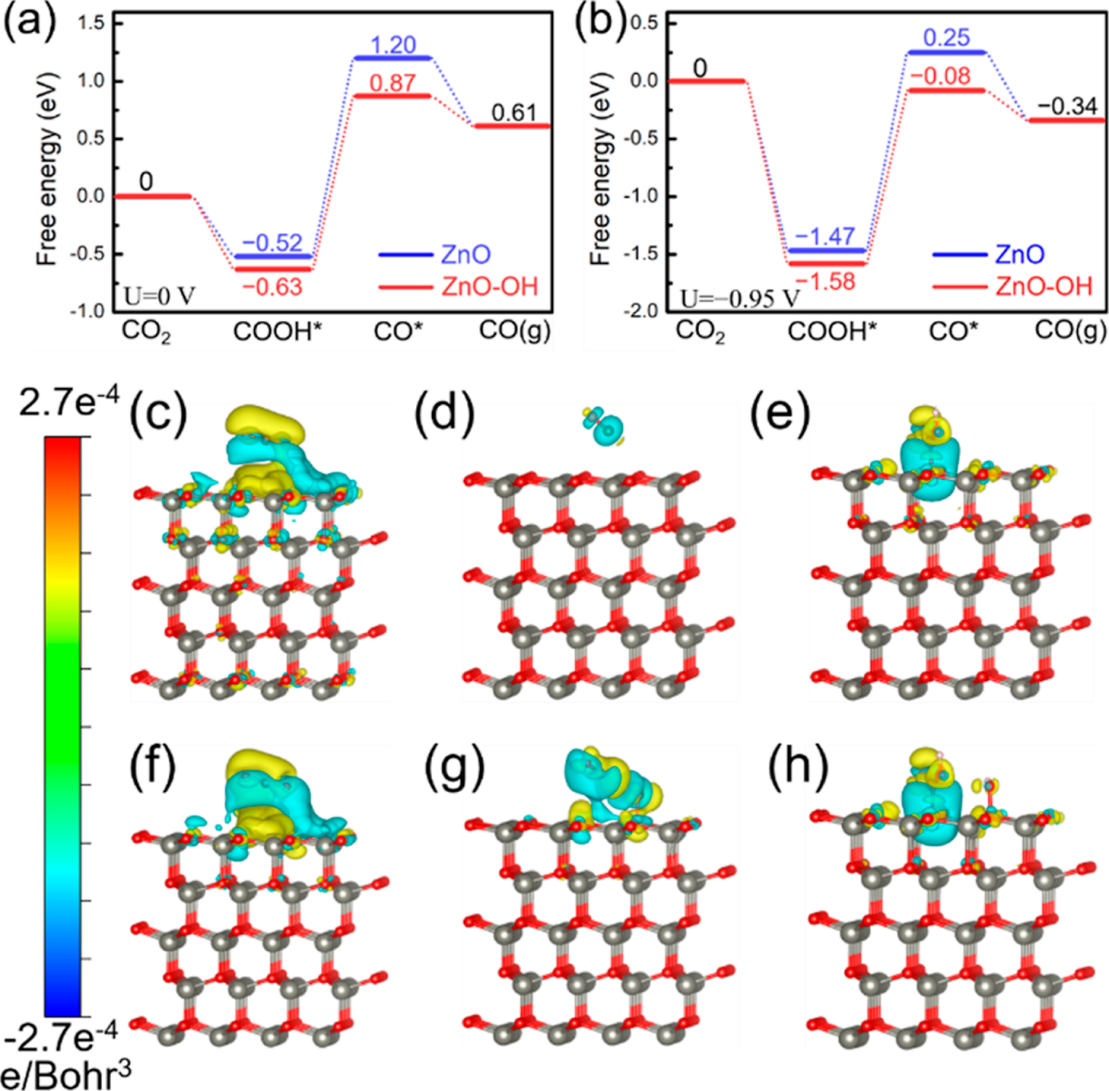
DFT studies of CO_2_ RR. Free-energy profiles for the (a) CO_2_ RR to CO at 0 and (b) −0.95 V (vs RHE) on the simulated models. Charge density difference for CO_2_, CO, and *COOH adsorbed on a (c–e) ZnO slab and a (f–h) ZnO–OH slab. The gray, red, brown, and pink spheres represent Zn, O, C, and H atoms, respectively.

The CO_2_ RR process for the ZnO and ZnO–OH models was studied by DFT calculations to illustrate the origin of the improved CO_2_ RR. The free-energy profiles at a potential of 0 V versus RHE for the three elementary steps and the two important intermediates (COOH* and CO*) in the CO_2_ RR process are shown in Figure 5a. The Δ*G* for the formation of COOH* over commercial ZnO and the ZnO–OH catalysts is −0.52 and −0.63 eV, respectively. The stronger stabilization of surface COOH* on the ZnO–OH could increase the selectivity for the desired product CO. Besides, the following dissociation of COOH* assisted by the proton-electron transfer to produce CO* and H_2_O is an endothermic and the RDS. To our excitement, Δ*G* increases by 1.72 and 1.5 eV on ZnO and ZnO–OH catalyst models, respectively, which means that the process of the CO* generation on the ZnO–OH slab is thermodynamically more favorable than that on the ZnO slab. As for the final step of CO desorption, the Δ*G* over the reference ZnO and the ZnO–OH catalyst is −0.59 and −0.26 eV, respectively. Such a relatively weak binding of CO* and above stronger stabilization of COOH* steer the electron and proton transfer to the formation of the CO product. Similar trends are also observed on the free-energy profiles at −0.95 V (vs RHE) (Figure 5b). The differential charge density for CO_2_, CO, and COOH* on the ZnO–OH and ZnO slabs was also calculated and is shown in Figure 5c–h. The charge accumulation and deficit between them and the corresponding surface were presented with yellow and cyan iso-surfaces. Through contrastive analysis, the charge density difference of CO on the ZnO–OH slab was more prominent than that on the ZnO slab. To summarize, the ZnO–OH slab stabilizes the key intermediates via electronic interactions, which in synergy leads to an enhanced CO selectivity. More importantly, the Gibbs free energy for the CO_2_ activation process on ZnO with two surficial −OH decreased by ca. 0.17 eV with respect to the ZnO with only one surficial −OH, which revealed that more surficial −OH coverage enhanced the adsorption of CO_2_ (Figure S22). These calculated results were in good agreement with the experimental observations that the ZnO–OH sample exhibited better selectivity for CO_2_ RR in comparison to the C–ZnO catalyst. Additionally, HER as a competing side reaction is also studied here (Figure S23). The stronger stability of H* on the surface could suppress HER effectively. It can be concluded that the HER is less active on ZnO–OH than that on the reference ZnO sample (−1.87 and −1.67 eV, respectively), suggesting that HER occurs more easily on ZnO without the surficial −OH coverage. Taken in all, the surface hydroxyls can not only facilitate the formation of COOH* and CO* via electronic interactions but also limit the undesired HER.

## 4 Conclusions

In summary, ZnO covered by surficial −OH groups was synthesized through a novel MOF-assisted method, which delicately optimize the interfacial microenvironment to promote the interfacial adsorption and activation of CO_2_. The synthesized −OH-rich ZnO presents a FE_CO_ maximum of 85% at −0.95 V versus RHE, which is one of the best records among the state-of-the-art ZnO-based catalysts. DFT calculations confirmed that the surface −OH first boosts the adsorption of CO_2_ at the interface and then promotes the generation of COOH* and CO* intermediates. Our findings revealed that tuning the interfacial microenvironment via the introduction of dioxide-philic functional groups is a promising way to achieve the global optimization via promotion of interfacial adsorption and activation of CO_2_, which paves a new way to rationally design future highly active electrocatalysts for eCO_2_ RR.

## Supporting Information Available

Additional characterizations, DFT results, and electrocatalytic testing curves (PDF)
