# Supplementary material for: Engineering the Interfacial Microenvironment via Surface Hydroxylation to Realize the Global Optimization of Electrochemical CO2 Reduction
Source: ACS Appl Mater Interfaces. 2022 Jul 11;14(28):32157–65. doi: 10.1021/acsami.2c09129 (PMC9305709; doi:10.1021/acsami.2c09129)
Supplement: Supplementary file 1 — am2c09129_si_001.pdf [file am2c09129_si_001.pdf]

Supporting Information

Engineering the Interfacial Microenvironment via Surface Hydroxylation to Realize the Global Optimization of Electrochemical CO_2 Reduction

Xu Han,[a,+] Ting Zhang,[a,b,+] Martí Biset-Peiró,[b] Xuan Zhang,[c] Jian Li,[d,*] Weiqiang Tang,[e,*] Pengyi Tang,[f] Joan Ramon Morante,[b,g] and Jordi Arbiol[a,h,*]

[a]Catalan Institute of Nanoscience and Nanotechnology (ICN2), CSIC and BIST, Campus UAB, Bellaterra, 08193, Barcelona, Catalonia, Spain

[b]Catalonia Institute for Energy Research (IREC), Jardins de les Dones de Negre 1, Sant Adrià del Besòs, 08930, Barcelona, Catalonia, Spain

[c]Department of Materials Engineering, KU Leuven, Leuven 3001, Belgium

[d]Laboratory of Renewable Energy Science and Engineering, Institute of Mechanical Engineering, EPFL, Station 9, 1015 Lausanne, Switzerland

[e]State Key Laboratory of Chemical Engineering and School of Chemical Engineering, East China University of Science and Technology, 200237 Shanghai, China

[f]State Key Laboratory of Information Functional Materials, 2020 X-Lab, Shanghai Institute of Microsystem and Information Technology, Chinese Academy of Sciences, 200050 Shanghai, China

[g]Department of Physics, Universitat de Barcelona, 08028, Barcelona, Catalonia, Spain

[h]ICREA, Pg. Lluís Companys 23, 08010 Barcelona, Catalonia, Spain

*Corresponding author:

E-mail: jian.li@epfl.ch

E-mail: wqtang@ecust.edu.cn

E-mail: arbiol@icrea.cat

[+]X.H. and T.Z. contributed equally to this paper.

Material Characterization. The X-ray diffraction patterns (XRD) were obtained through a Bruker D4 X-ray powder diffractometer using Cu Kα radiation (1.54184 Å). Field emission scanning electron microscopy (FESEM) images were collected on a FEI Magellan 400 L scanning electron microscope. The transmission electron microscopy (TEM) and high angle annular dark field scanning TEM (HAADF-STEM) images were obtained in a Tecnai F20 field emission gun microscope with a 0.19 nm point-to-point resolution at 200 kV equipped with an embedded Quantum Gatan Image Filter for EELS analyses. Images have been analyzed by means of Gatan Digital Micrograph software. X-ray photoelectron spectroscopy (XPS) was performed on a Phoibos 150 analyser (SPECS GmbH, Berlin, Germany) in ultra-high vacuum conditions (base pressure 4×10^{-10} mbar) with a monochromatic aluminum Kα X-ray source (1486.74 eV). Binding energies (BE) were determined using the C 1s peak at 284.5 eV as a charge reference. Brunauer-Emmett-Teller (BET) surface areas were measured using nitrogen adsorption at 473 K.

Calculation Method:

Details concerning the calculation of Faradaic Efficiency (FE) is shown as below.[1]

The partial current density for a given gas product was calculated as below:

$$j_i = x_i \times V \times \frac{n_i F P_0}{RT} \times (electrode\ area)^{-1} \quad (1)$$

Where x_i is the volume fraction of certain product determined by online GC referenced to calibration curves from three standard gas samples, v is the flow rate, n_i is the number of electrons involved, P_0= 101.3 kPa, F is the Faraday constant, and R is the gas constant.

The corresponding FE at each potential is calculated by

$$FE = \frac{j_i}{j} \times 100\% \quad (2)$$

DFT Calculations:

The spin-polarized DFT calculations with projector augmented wave (PAW) method were performed using the Vienna Ab initio Simulation Package (VASP) code. The generalized gradient approximation of Perdew-Burke-Ernzerhof (PBE) with van der Waals correlation was employed to optimize the geometric structures. The convergence criteria were 0.05 eV/ Å in force and 1×10^{-5} eV in energy and the plane wave cutoff was 550 eV. The Monkhorst–Pack mesh *k*-point grids was 3×3×1 for all models. All the vacuum thicknesses were higher than 15 Å.

The binding energy (E_{b}) of CO_2 with ZnO or ZnOH slab was defined as:

$$E_{\mathrm{b}} = E_{\mathrm{total}} - E_{\mathrm{slab}} - E_{\mathrm{CO_2}} \tag{3}$$

where $E_{\mathrm{CO_2}}$, E_{slab} and E_{total} are the energies of the CO_2 molecule in gas phase, the corresponding clean slab system (clean ZnO and ZnOH slab), and the total energies of the adsorbed system, respectively.

The whole process of CO_2 electrochemical reduction to CO mainly includes the following three steps:

$$CO_2(g) + * + H^+ + e^- \leftrightarrow COOH^* \text{ (Activation process)} \tag{4}$$

$$COOH^* + H^+ + e^- \leftrightarrow CO^* + H_2O(l) \text{ (Surface reaction)} \tag{5}$$

$$CO^* \leftrightarrow CO(g) + * \text{ (Desorption process)} \tag{6}$$

Where the *, COOH* and CO* represent free site, adsorption state of COOH and CO, respectively. The (g) represent the gas phase. The reaction free energies of each steps were calculated by following formula:

$$G = E_{\mathrm{DFT}} + E_{\mathrm{ZPE}} - TS + E_{\mathrm{sol}} \tag{7}$$

Where E_{DFT} is the DFT calculated energy, E_{ZPE} is the zero-point energy, *T* (=298.15 K) is temperture, *S* is the entropy, and E_{sol} is solvation correction and for CO* was stabilized by 0.1 eV and COOH* by 0.25 eV.

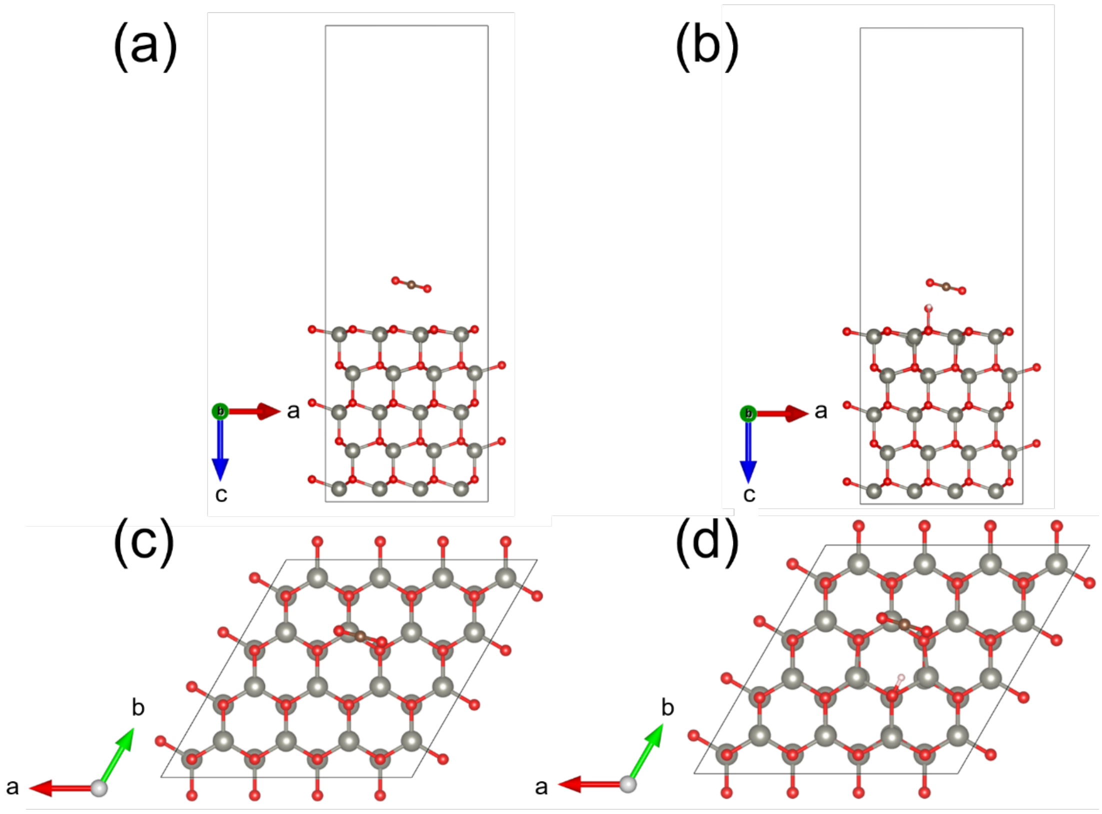

Figure S1. Side and top view models for the (a, c) ZnO slab and (b, d) ZnO-OH slab. (The grey, red, and brown spheres represent Zn, O and C atoms, respectively)

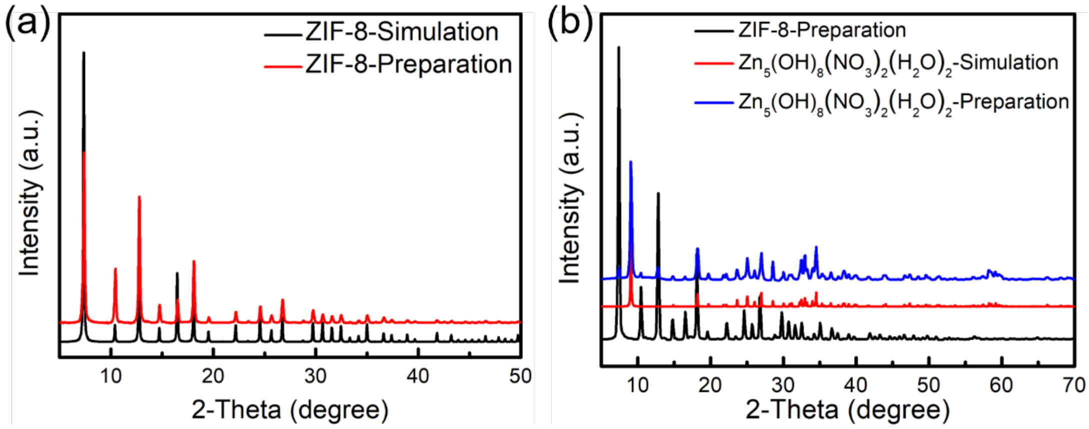

Figure S2. XRD patterns of (a) ZIF-8 and (b) corresponding hydroxide intermediate.

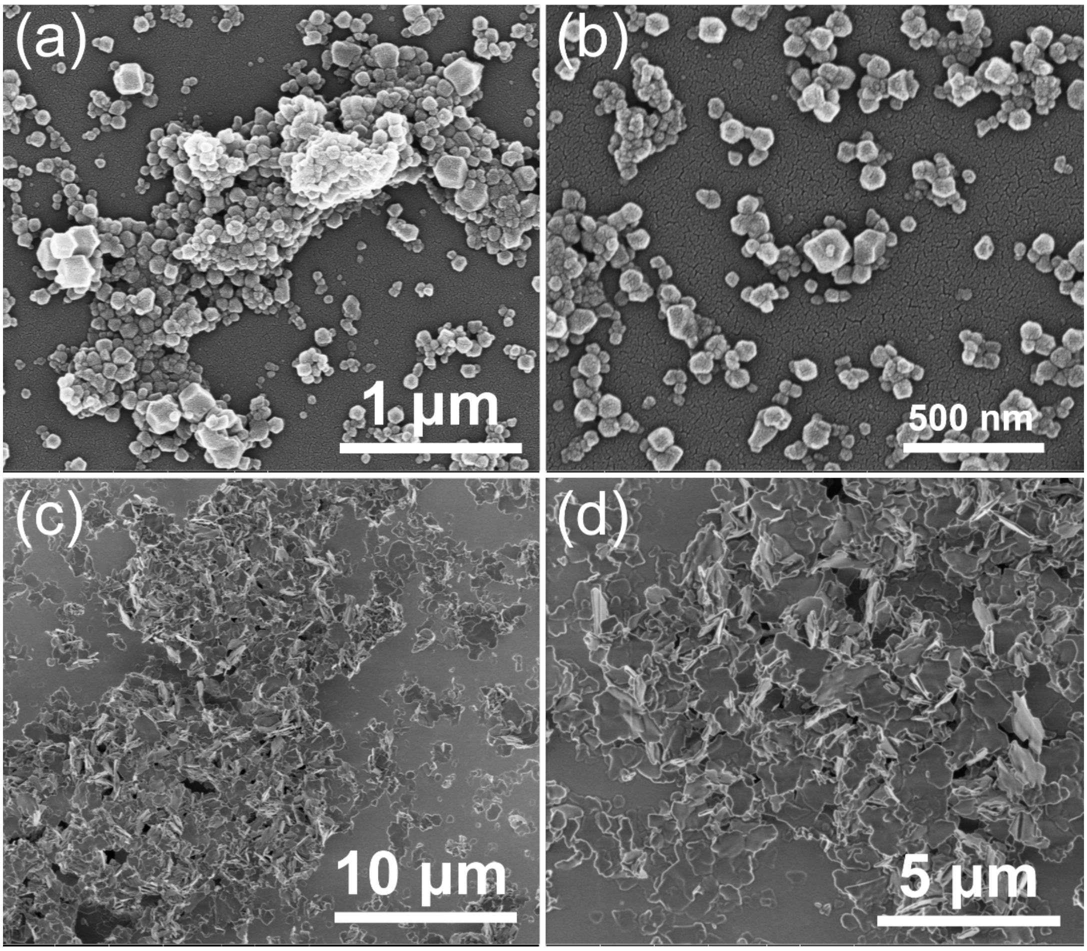

Figure S3. SEM images of (a, b) ZIF-8 and (c, d) $Zn_5(OH)_8(NO_3)_2$ $(H_2O)_2$.

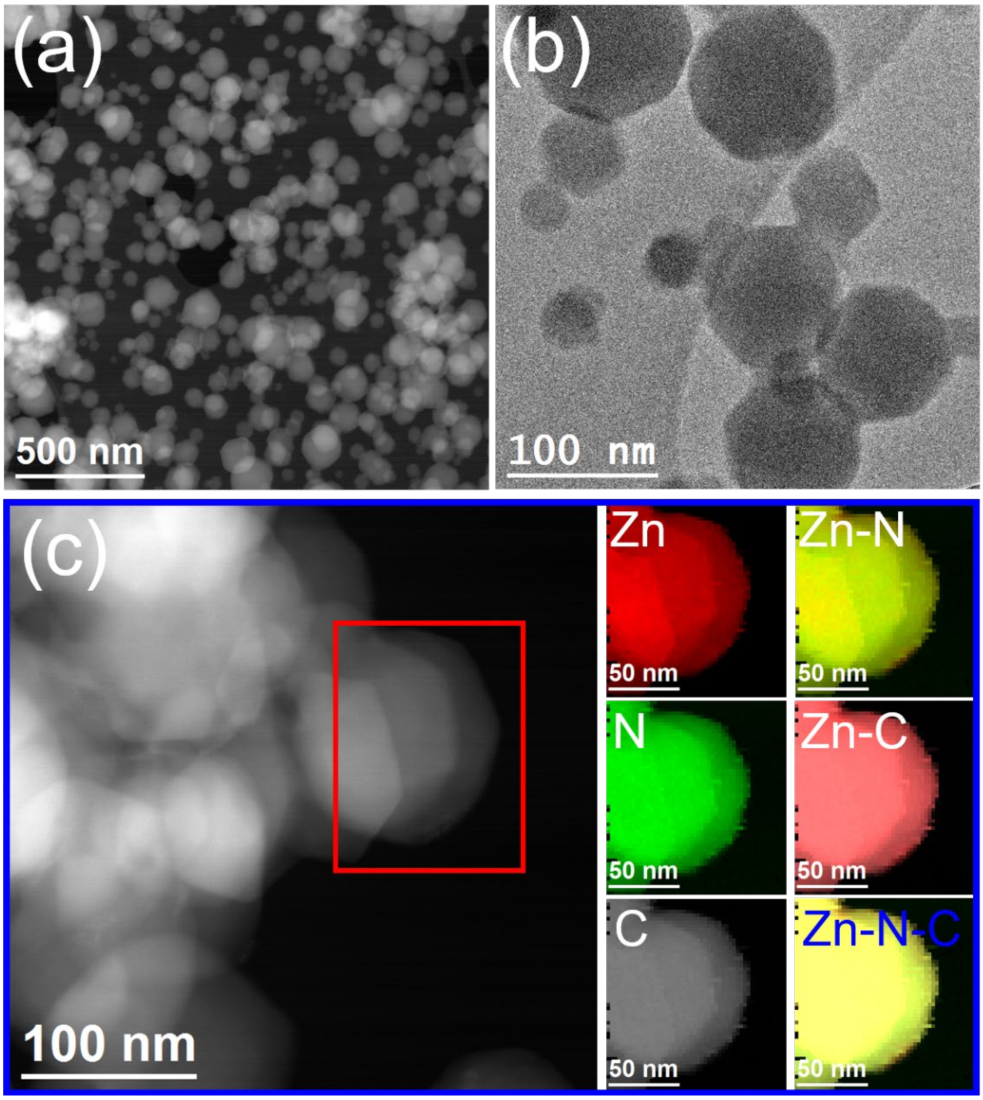

Figure S4. (a) HAADF-STEM, (b) BF TEM, (c) HAADF-STEM image of ZIF-8 and representative EELS chemical composition maps obtained from the red squared area of the STEM micrograph. Individual Zn $L_{2,3}$-edges at 1020 eV (red), N K-edges at 401 eV (green), and C K-edges at 285 eV (grey) as well as composites of Zn-N, Zn-C and Zn-N-C.

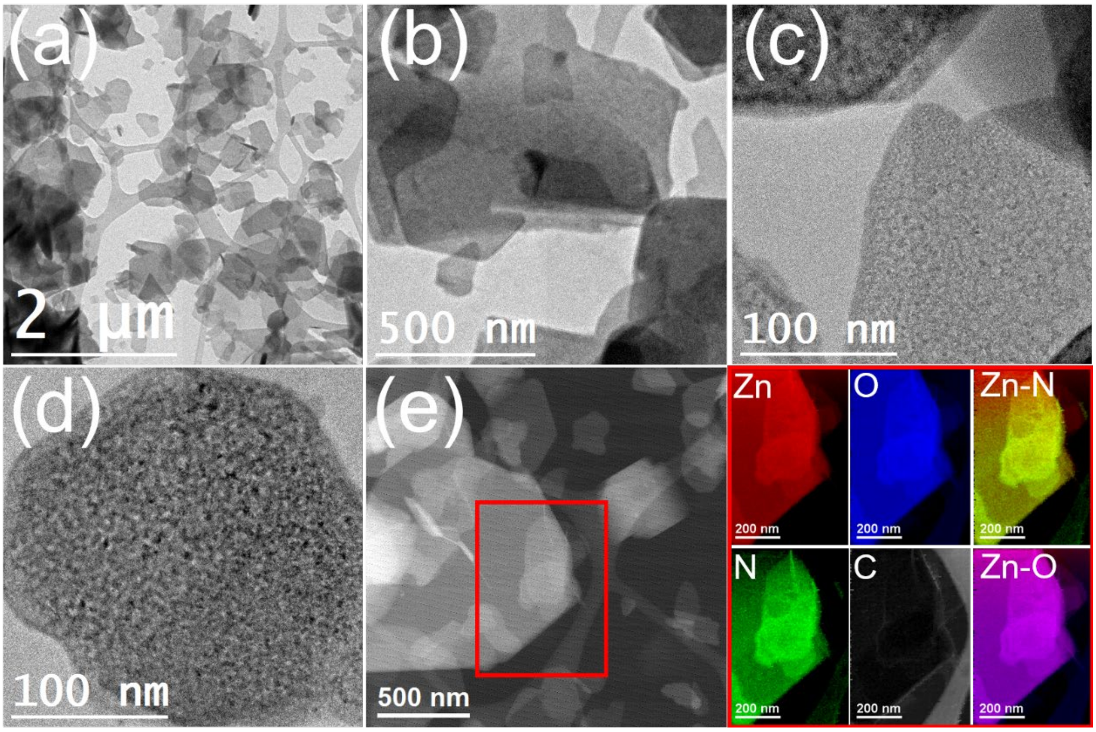

Figure S5. (a-d) BF TEM and HAADF-STEM image of $Zn_5(OH)_8(NO_3)_2(H_2O)_2$ and (e) representative EELS chemical composition maps obtained from the red squared area of the STEM micrograph. Individual Zn $L_{2,3}$-edges at 1020 eV (red) N K-edges at 401 eV (red), O K-edges at 532 eV (blue) and C K-edges at 285 eV (grey) as well as composites of Zn-N and Zn-O.

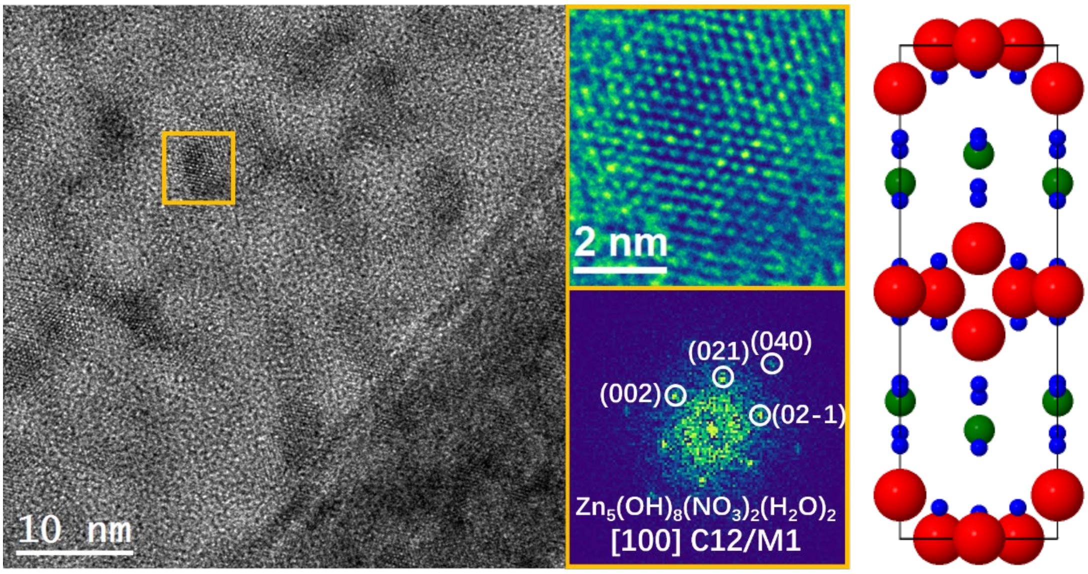

Figure S6. HRTEM micrograph of $Zn_5(OH)_8(NO_3)_2(H_2O)_2$ sample and detail of the orange squared region and its corresponding power spectrum as well as unit cell illustration of the $Zn_5(OH)_8(NO_3)_2(H_2O)_2$ (Zn, N and O atoms are represented in red, green and blue, respectively).

Figure S6 shows a HRTEM micrograph of a nanoparticle. Detail of the orange squared region and its corresponding power spectrum which reveals that this sample has a crystal phase that is in agreement with the $Zn_5(OH)_8(NO_3)_2(H_2O)_2$ monoclinic phase (space group =IA3-) with a=19.4800 Å, b=6.2380 Å, and c=5.5170 Å. From the crystalline domain in **Figure S6**, the $Zn_5(OH)_8(NO_3)_2(H_2O)_2$ lattice fringe distances were measured to be 0.282 nm, 0.276 nm, 0.162 nm and 0.276 nm, at 60.33º, 92.41º and 120.44º which could be interpreted as the monoclinic $Zn_5(OH)_8(NO_3)_2(H_2O)_2$ phase, visualized along its [100] zone axis.

Table S1. Comparison between the experimental and the theoretical bulk plane spacing distances and angles between planes.

Spot	Experimental (nm)	$Zn_5(OH)_8(NO_3)_2(H_2O)_2$ (C12/M1) [100]
1	0.282	0.275 (002)
2	0.276 (60.33º vs Spot 1)	0.271 (60.48º) (021)
3	0.162 (92.41º vs Spot 1)	0.156 (90.00º) (040)
4	0.276 (120.44º vs Spot 1)	0.271 (119.52º) (02-1)

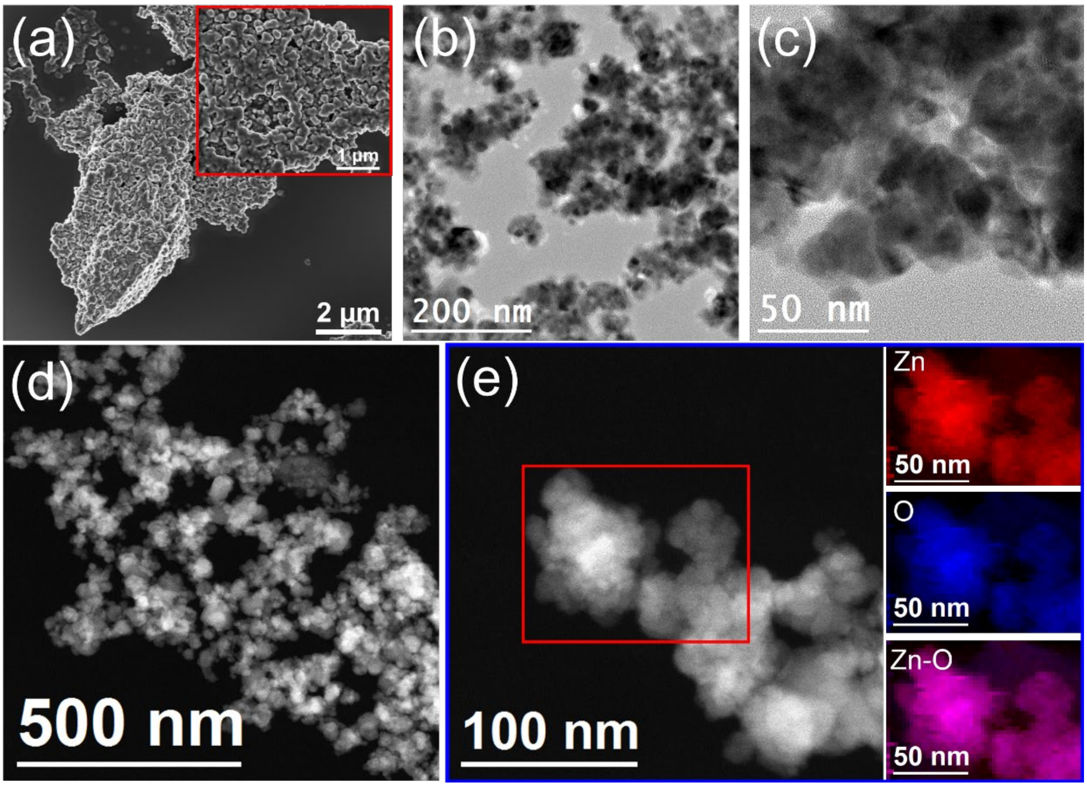

Figure S7. (a-d) BF TEM and HAADF-STEM image of D-ZnO and (e) representative EELS chemical composition maps obtained from the red squared area of the STEM micrograph. Individual Zn $L_{2,3}$-edges at 1020 eV (red) and O K-edges at 532 eV (blue) as well as composites of Zn-O.

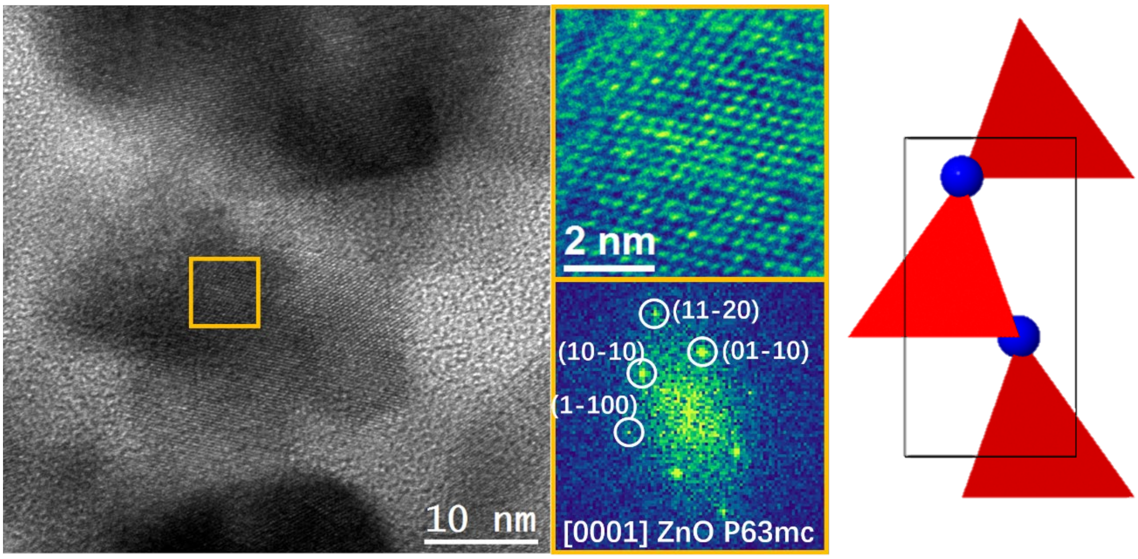

Figure S8. HRTEM micrograph of D-ZnO sample and detail of the orange squared region and its corresponding power spectrum as well as 1*1*1 unit crystal model of ZnO. (Zn and O atoms are represented in red and blue, respectively)

Figure S8 shows a HRTEM micrograph taken from the nanoparticle squared in orange. Detail of the orange squared region and its corresponding power spectrum which reveals that this nanoparticle has a crystal phase that could be in agreement with the ZnO hexagonal phase (space group =P63mc) with a=b=3.2900 Å and c=5.3000 Å. From the crystalline domain in **Figure S8**, the ZnO lattice fringe distances were measured to be 0.266 nm, 0.284 nm, 0.163 nm and 0.275 nm, at 59.01º, 90.62º and 121.94º, which could be interpreted as the hexagonal ZnO phase, visualized along its [0001] zone axis.

Table S2. Comparison between the experimental and the theoretical bulk plane spacing distances and angles between planes.

Spot	Experimental (nm)	ZnO (P63mc) [0001]
1	0.266	0.285 (1-100)
2	0.284 (59.01º vs Spot 1)	0.285 (60.00º) (10-10)
3	0.163 (90.62º vs Spot 1)	0.165 (90.00º) (11-20)
4	0.275 (121.94º vs Spot 1)	0.285 (120.00º) (01-10)

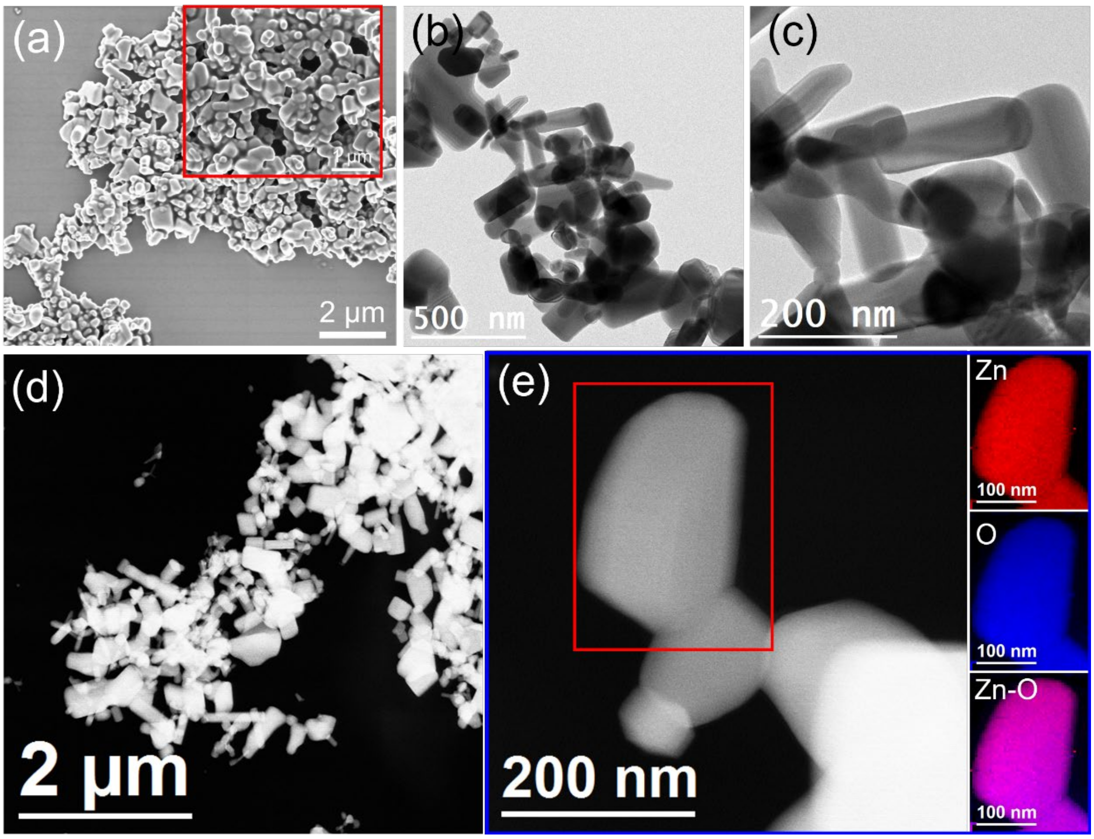

Figure S9. (a-d) SEM, BF TEM and HAADF STEM image of C-ZnO and (e) representative EELS chemical composition maps obtained from the red squared area of the STEM micrograph. Individual Zn $L_{2,3}$-edges at 1020 eV and O K-edges at 532 eV (blue) as well as composites of Zn-O.

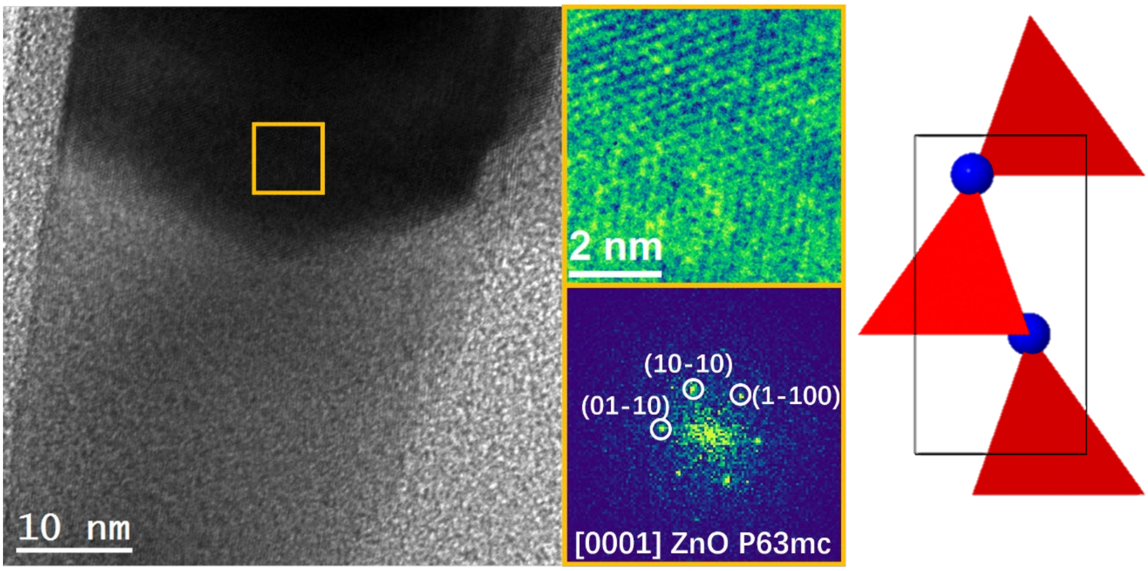

Figure S10. HRTEM micrograph of C-ZnO sample and detail of the orange squared region and its corresponding power spectrum as well as 1*1*1 unit crystal model of ZnO. (Zn and O atoms are represented in red and blue, respectively)

Figure S10 shows a HRTEM micrograph taken from the nanoparticle squared in orange. Detail of the orange squared region and its corresponding power spectrum which reveals that this nanoparticle has a crystal phase that could be in agreement with the ZnO hexagonal phase (space group =P63mc) with a=b=3.2900 Å and c=5.3000 Å. From the crystalline domain in **Figure S10**, the ZnO lattice fringe distances were measured to be 0.283 nm, 0.282 nm and 0.284 nm, at 63.03º and 122.66º, which could be interpreted as the hexagonal ZnO phase, visualized along its [0001] zone axis.

Table S3. Comparison between the experimental and the theoretical bulk plane spacing distances and angles between planes.

Spot	Experimental (nm)	ZnO (P63mc) [0001]
1	0.283	0.285 (01-10)
2	0.282 (63.03º vs Spot 1)	0.285 (60.00º) (10-10)
3	0.284 (122.66º vs Spot 1)	0.285 (90.00º) (1-100)

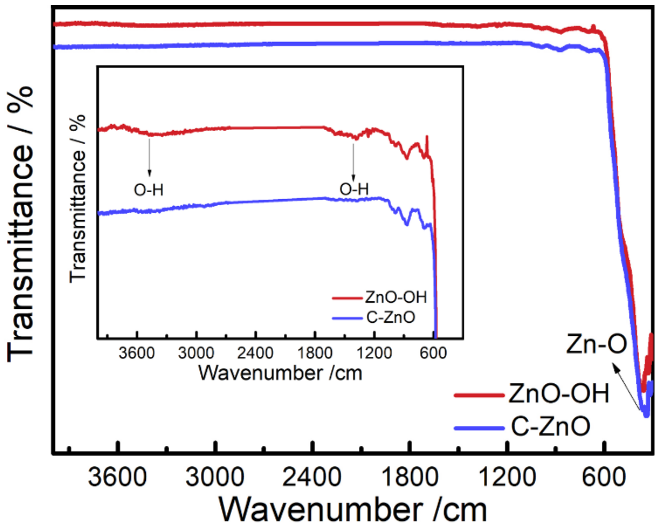

Figure S11. FTIR spectrum of ZnO-OH and C-ZnO (inset: the larger FTIR image).

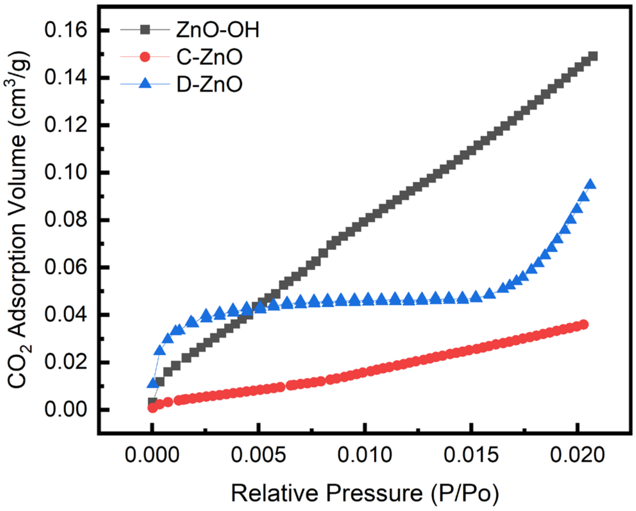

Figure S12. CO_2 adsorption isotherm recorded on different samples at 298 K.

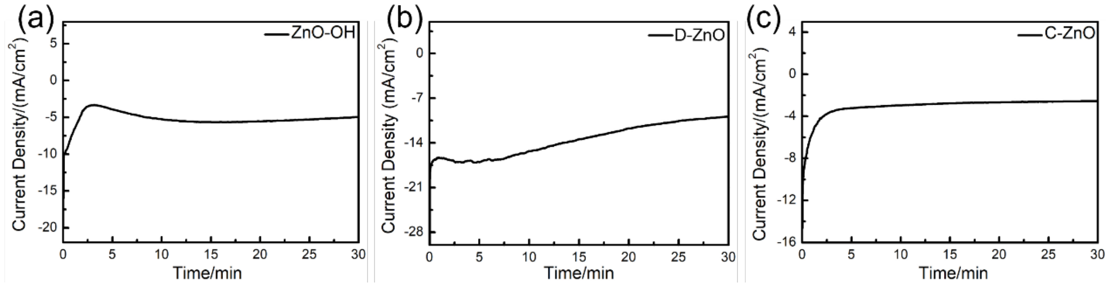

Figure S13. Electrode current recorded during reduction of (a) ZnO-OH, (b) D-ZnO and (c) C-ZnO samples at −0.70 V vs. RHE in 0.5 M $NaHCO_3$ purged with Ar gas.

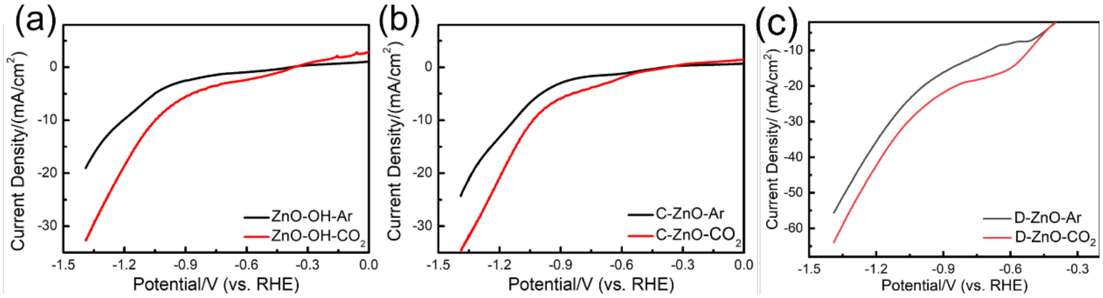

Figure S14. Linear sweep voltammetry (LSV) comparison for (a) ZnO-OH, (b) C-ZnO and (c) D-ZnO.

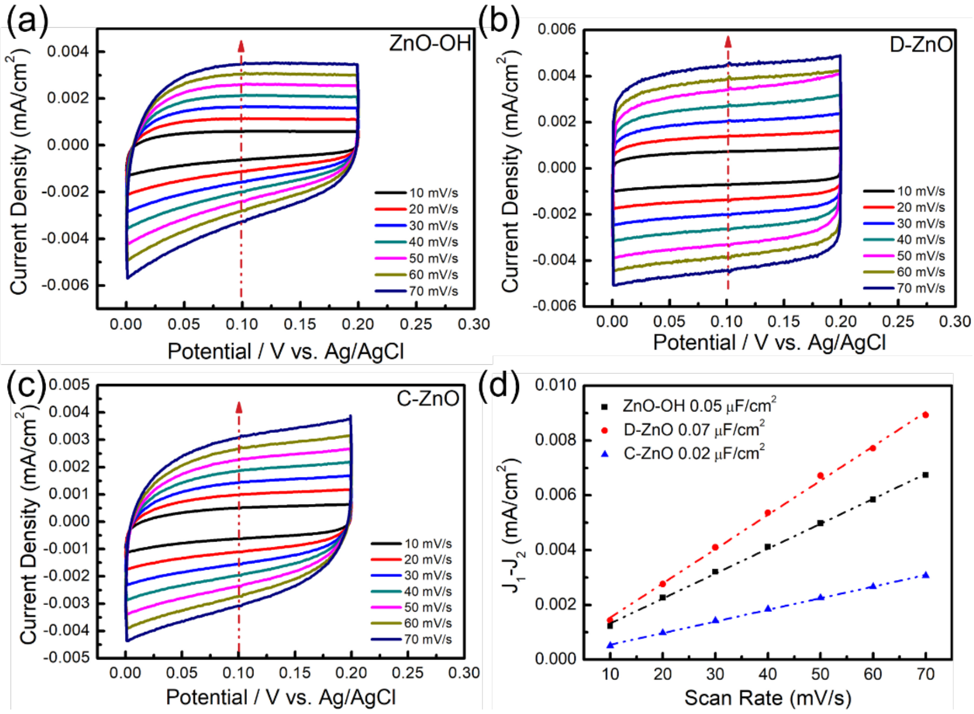

Figure S15. Cyclic voltammograms curves for (a) ZnO-OH, (b) D-ZnO, (c) C-ZnO and (d) Plots of the current density vs. scan rate for ZnO-OH, D-ZnO and C-ZnO electrodes.

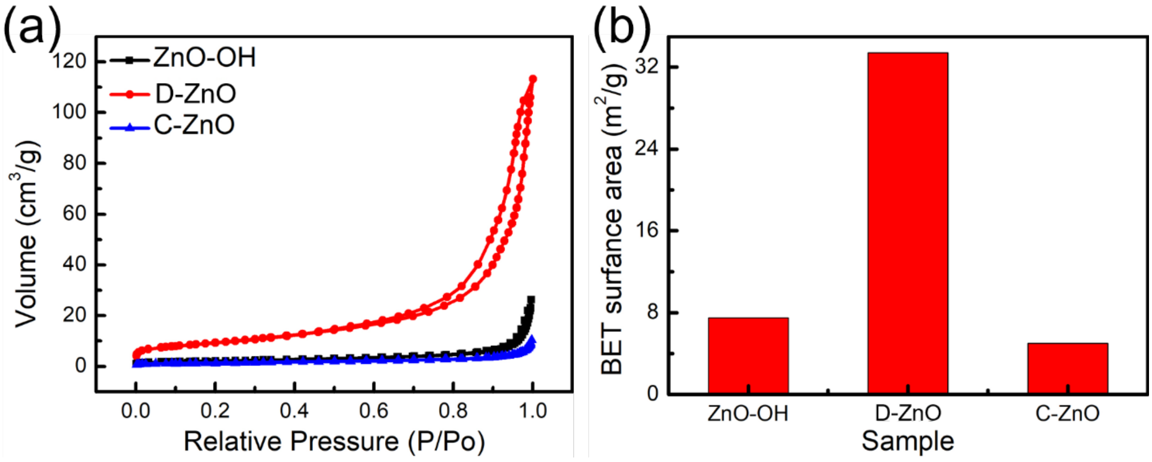

Figure S16. (a) N_2 adsorption and desorption isotherm and (b) BET surface areas for ZnO-OH, D-ZnO and C-ZnO.

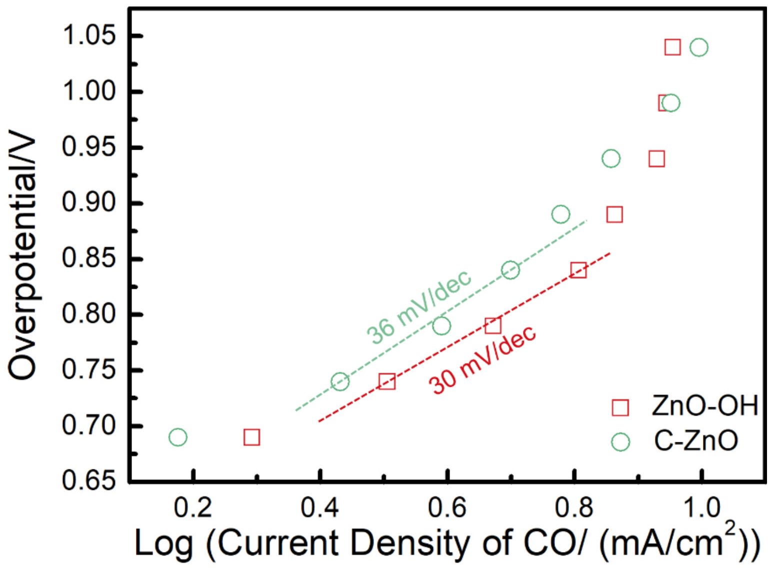

Figure S17. Tafel Slope of ZnO-OH and C-ZnO.

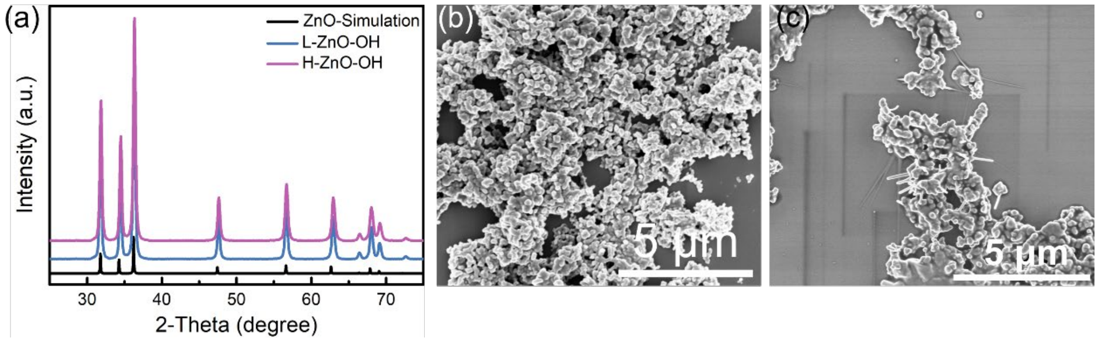

Figure S18. (a) XRD pattens and SEM images of (b) L-ZnO-OH and (c) H-ZnO-OH.

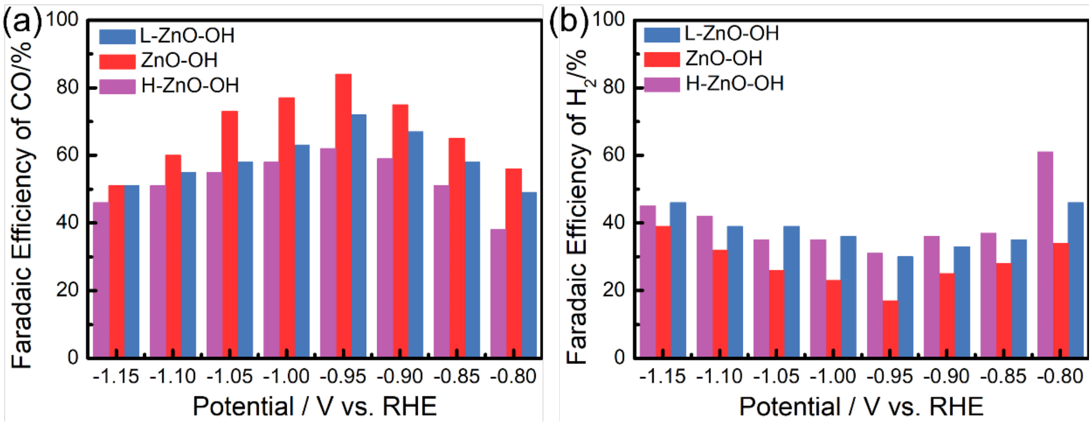

Figure S19. FE of (a) CO and (b) H_2 at various potentials on L-ZnO-OH, ZnO-OH and H-ZnO-OH.

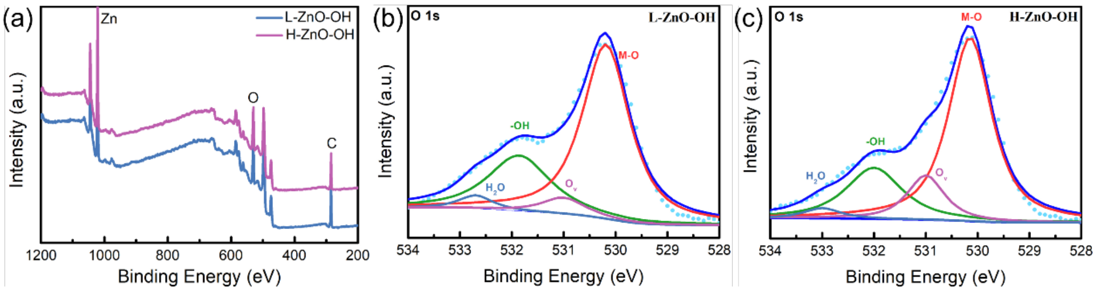

Figure S20. (a) XPS survey spectra, high resolution XPS spectra of O 1s for (b) L-ZnO-OH and (c) H-ZnO-OH.

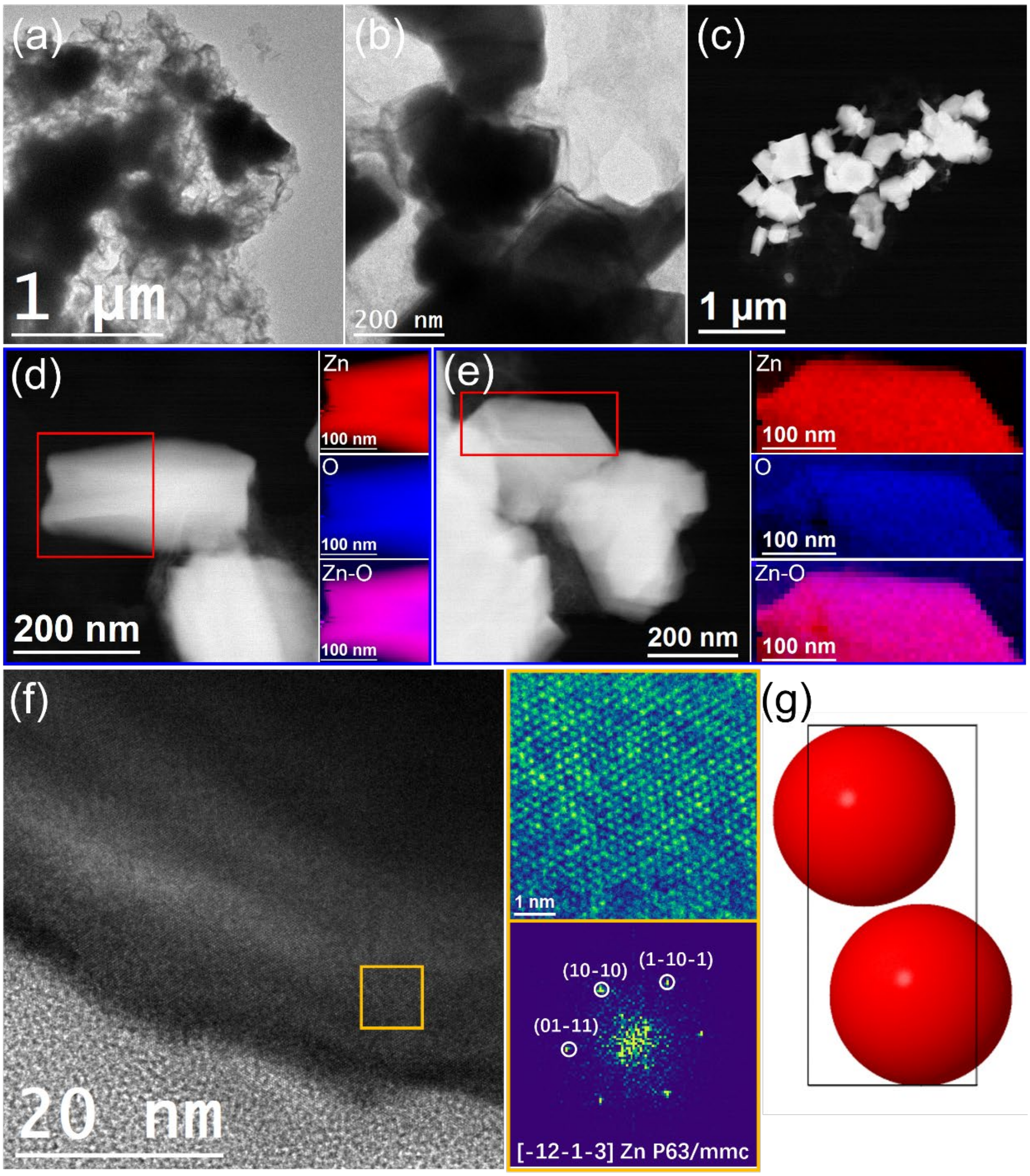

Figure S21. Morphological characterization of ZnO-OH sample after stability test. (a and b) BF TEM images, (c) HAADF STEM image. (d and e) HAADF STEM image and their representative EELS chemical composition maps obtained from the red squared area of the STEM micrograph. Individual Zn $L_{2,3}$-edges at 1020 eV (red), O K-edges at 532 eV (blue) and composite of Zn-O. (f) is HRTEM image and corresponding to FFT spectrum. (g) is its corresponding power spectrum as well as 1*1*1 unit crystal model of Zn. (Zn atoms are represented in red)

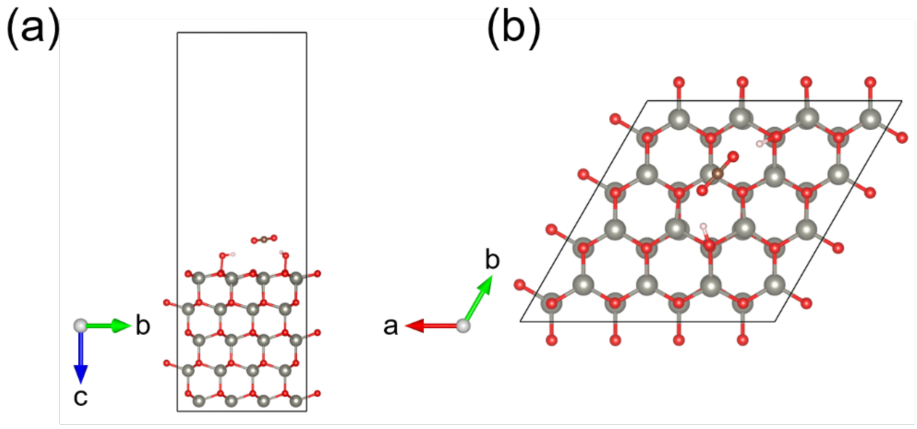

Figure S22. The (a) side and (b) top view of models for ZnO-OH slab with two -OH (The grey, red, and brown spheres represent Zn, O and C atoms, respectively).

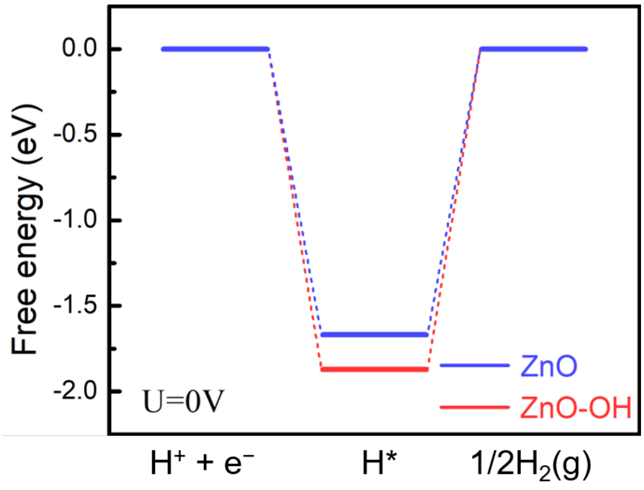

Figure S23. DFT Studies of CO_2 RR. Free energy profiles for the HER to CO at 0 V on simulated models.

Table S4. Faradaic Efficiency (CO) of the reported ZnO-based electrocatalysts for CO_2 electroreduction

Catalyst	Product	FE(CO)	Potential	Reference
ZnO nanosheet@Zn	CO	85 %	−2.0 V vs. Ag/AgCl	[2]
ZnO with vacancy	CO	83 %	−1.10 V vs. RHE	[3]
ZnO	Syngas	32 %	−1.0 V vs. RHE	[4]
ZnO with vacancy	CO	58 %	−0.79 V vs. RHE	[5]
ZnO	CO	54 %	−1.40 V vs. RHE	[6]
Ce-doped ZnO	CO	88 %	−1.0 V vs. RHE	[7]
**ZnO-OH**	**CO**	**85%**	**−0.95 V vs. RHE**	**This work**

References

(1) Zheng, T.; Jiang, K.; Ta, N.; Hu, Y.; Zeng, J.; Liu, J.; Wang, H. Large-Scale and Highly Selective CO_2 Electrocatalytic Reduction on Nickel Single-Atom Catalyst. *Joule* **2019,** *3* (1), 265-278.

(2) Xiang, Q.; Li, F.; Wang, J.; Chen, W.; Miao, Q.; Zhang, Q.; Tao, P.; Song, C.; Shang, W.; Zhu, H.; Deng, T.; Wu, J. Heterostructure of ZnO Nanosheets/Zn with a Highly Enhanced Edge Surface for Efficient CO_2 Electrochemical Reduction to CO. *ACS Appl. Mater. Interfaces* **2021,** *13* (9), 10837-10844.

(3) Geng, Z.; Kong, X.; Chen, W.; Su, H.; Liu, Y.; Cai, F.; Wang, G.; Zeng, J. Oxygen Vacancies in ZnO Nanosheets Enhance CO_2 Electrochemical Reduction to CO. *Angew. Chem. Int. Ed.* **2018,** *57* (21), 6054-6059.

(4) Daiyan, R.; Lovell, E. C.; Huang, B.; Zubair, M.; Leverett, J.; Zhang, Q.; Lim, S.; Horlyck, J.; Tang, J.; Lu, X.; Kalantar-Zadeh, K.; Hart, J. N.; Bedford, N. M.; Amal, R. Uncovering Atomic-Scale Stability and Reactivity in Engineered Zinc Oxide Electrocatalysts for Controllable Syngas Production. *Adv. Energy Mater.* **2020,** *10* (28), 2001381.

(5) Meng, N.; Huang, Y.; Liu, Y.; Yu, Y.; Zhang, B. Electrosynthesis of Urea from Nitrite and CO_2 over Oxygen Vacancy-Rich ZnO Porous Nanosheets. *Cell Rep. Phys. Sci.* **2021,** *2* (3), 100378.

(6) Xue, L.; Zhang, A.; Wu, J.; Wang, Q.; Liu, Y.; Zhao, Y.; Liu, S.; Liu, Z.; Li, P.; Zeng, S. Surface Modification and Reconstruction of ZnO Hollow Microspheres for Selective Electroreduction of CO_2 to CO. *J. Alloys. Compd.* **2021,** *882*, 160703.

(7) Ren, X.; Gao, Y.; Zheng, L.; Wang, Z.; Wang, P.; Zheng, Z.; Liu, Y.; Cheng, H.; Dai, Y.; Huang, B. Oxygen Vacancy Enhancing CO_2 Electrochemical Reduction to CO on Ce-Doped ZnO Catalysts. *Surf. Interfaces* **2021,** *23*, 100923.
